# Altered development of fetal liver perfusion in pregnancies with pregestational diabetes

**DOI:** 10.1371/journal.pone.0211788

**Published:** 2019-03-13

**Authors:** Agnethe Lund, Cathrine Ebbing, Svein Rasmussen, Torvid Kiserud, Mark Hanson, Jörg Kessler

**Affiliations:** 1 Department of Obstetrics and Gynecology, Haukeland University Hospital, Bergen, Norway; 2 Research Group for Pregnancy, Fetal Development and Birth, Department of Clinical Science, University of Bergen, Norway; 3 Institute of Developmental Sciences, University of Southampton, Southampton, United Kingdom; University of Insubria, ITALY

## Abstract

**Background:**

Pregestational diabetes is associated with fetal macrosomia, and umbilical perfusion of the fetal liver has a role in regulating fetal growth. We therefore hypothesized that pregestational diabetes alters fetal liver blood flow depending on degree of glycemic control.

**Methods:**

In a prospective study, 49 women with pregestational diabetes underwent monthly ultrasound examinations during 24–36 gestational weeks. Blood flow was determined in the umbilical vein, ductus venosus and portal vein, and blood velocity was measured in the left portal vein, the latter reflecting the watershed between splanchnic and umbilical flow. The measurements were compared with reference values by z-score statistics, and the effect of HbA_1c_ assessed.

**Results:**

The umbilical venous flow to the liver (*z*-score 0.36, p = 0.002), total venous liver flow (*z*-score 0.51, p<0.001) and left portal vein blood velocity (*z*-score 0.64, p<0.001), were higher in the study group. Normalized portal venous flow was lower (*z*-score -0.42, p = 0.002), and normalized total venous liver flow tended to be lower after 30 gestational weeks (*z*-score -0.54, p = 0.047) in the diabetic pregnancies compared with reference values from a low-risk population. The left portal vein blood velocity was positively, and the portal fraction of total venous liver flow negatively correlated with first trimester HbA_1C_.

**Conclusions:**

In spite of increased umbilical blood distribution to the fetal liver, graded according to glycemic control, the total venous liver flow did not match third trimester fetal growth in pregnancies with pregestational diabetes, thus contributing towards increased perinatal risks and possibly altered liver function with long-term metabolic consequences.

## Introduction

Pregnancies complicated by pregestational diabetes mellitus (PGDM) are associated with increased perinatal morbidity and mortality [1], and fetal macrosomia is related to these adverse neonatal outcomes [2]. Fetal hyperglycemia and hyperinsulinemia can cause accelerated fetal growth [3] even with HbA_1C_ levels within the recommended range [4], and this makes clinical surveillance in diabetic pregnancies challenging [5].

The liver has been called “the metabolic brain” of the fetus [6], controlling the distribution and utilization of nutrients from the placenta. Nutrient access and fetal liver blood flow act both independently and together to influence fetal growth and body composition [7, 8]. The fetal liver has two sources of venous supply; well-oxygenated blood from the placenta through the umbilical vein being the main source, and low-oxygenated blood from visceral organs through the portal vein. The distribution of the nutrient rich umbilical venous blood to the liver has been suggested to be a mechanism for regulation of fetal growth [9]. This is based on experimental studies showing that increasing liver flow from the umbilical vein leads to higher cell proliferation in the liver, heart, skeletal muscle and kidneys in fetal lamb [9]. In addition, studies of human low-risk pregnancies have shown that larger fetal size is associated with higher umbilical venous liver flow as a response to maternal glucose intake [10]. Also, higher umbilical venous flow to the liver is associated with newborn adiposity [11].

In studies of macrosomic fetuses in non-diabetic pregnancies, umbilical- and total venous liver flow was higher during the 2^nd^ and 3^rd^ trimester, including when normalized for estimated fetal weight [7, 12]. This indicates that increased umbilical venous flow led to augmented fetal growth in pregnancies without diabetes. In low-risk pregnancies, the portal venous contribution to the liver increases throughout gestation, and the same pattern is observed in macrosomic non-diabetic fetuses [7]. However, although the fetal liver is larger [13] and macrosomic growth is frequent in diabetic pregnancies [4], umbilical venous flow normalized for fetal weight, is lower [13, 14].

Fetal liver gene expression in baboons is different in the left and right liver lobes [15], and this is ascribed to the specific venous perfusion pattern during fetal life. Thus, fetal hemodynamic development might influence liver function and be part of a pathway regulating intrauterine growth, with possible long-term consequences [7, 12].

In diabetic pregnancies, fetal liver size measured by ultrasound is greater than in low-risk pregnancies and liver volume positively correlates with maternal HbA_1C_ [13]. Experimental studies in pigs showed that diabetes induces fetal liver hyperplasia [16], the fetal liver protein synthesis and glycogen reserves increase [16], and total body fat percentage is higher than in non-diabetic controls [17]. In human stillborn neonates of diabetic mothers, hepatic steatosis is prevalent and more severe than in stillborn of non-diabetic pregnancies [18].

Fetuses of women with PGDM have greater risk of later diabetes independently of genetic factors [19], possibly mediated through epigenetic mechanisms. It has been suggested that the human fetal strategy to prioritize fat deposition for neonatal survival evolved under conditions where high glycemic diets were not available; but with their currently widespread consumption, these mechanisms enhance fetal fat deposition [20]. As both diabetes [21] and chronic liver disease [22] are becoming increasingly prevalent, and there is currently much interest in the developmental origins of these conditions, studies of factors such as maternal diabetes on fetal liver development are called for as a basis for informing preventive strategies [23]. We therefore aimed to determine the fetal liver blood flow in PGDM pregnancies in a prospective longitudinal study and present the longitudinal development of venous liver blood flow during the second half of PGDM pregnancies.

## Materials and methods

**Fig 1:**
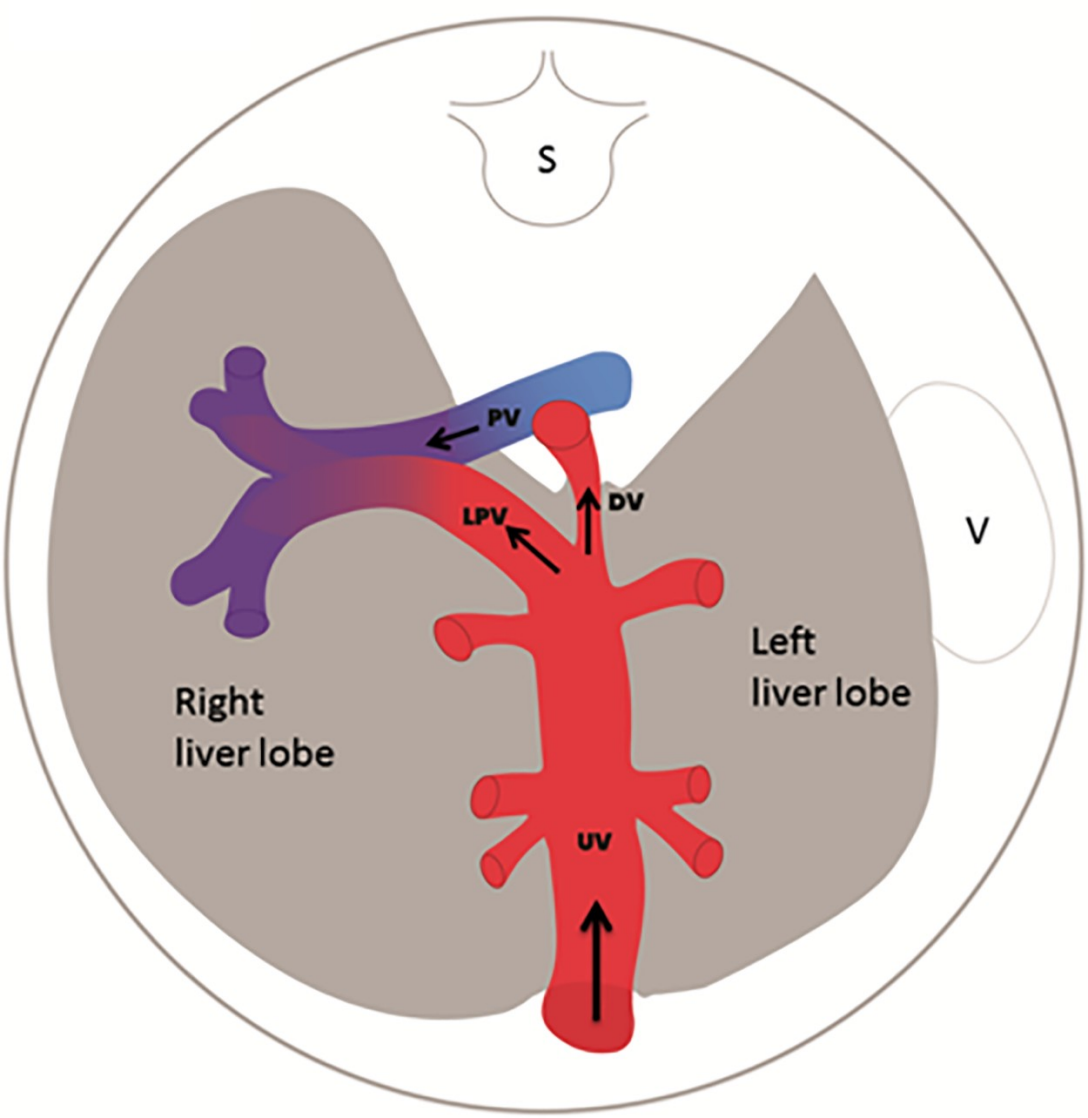
Venous supply to the fetal liver. Cross section of the fetal abdomen with black arrows indicating physiological blood flow directions in the fetal liver (grey). Typically, well-oxygenated umbilical blood (red) blends in with deoxygenated portal blood (blue) to feed the right liver lobe; UV, umbilical vein; DV, ductus venosus; LPV, Left portal vein; PV, portal vein; S, spine; V, stomach.

The present prospective longitudinal observational study is part of a larger project investigating fetal hemodynamics in pregnancies with PGDM. The study protocol was approved by the Regional Committee for Medical and Health Research Ethics (REK vest 2011/2030). We have reported the development of the umbilical venous and ductus venosus flows in this population [14]. Here we present data on the development of the venous supply to the fetal liver in PGDM pregnancies. We have used the left portal vein blood velocity as a marker of the watershed between the portal and umbilical venous contributions (Fig 1).

### Subjects

All women in our region, with PGDM in pregnancy, are referred to our tertiary center at Haukeland University Hospital for multidisciplinary follow-up. All women with singleton pregnancies and PGDM who presented at our clinic between August 2013 and May 2016 were invited to participate. Fifty-two women (74% of those invited) gave written consent: 44 participants had type 1 diabetes mellitus (DM) and 8 had type 2 DM of which all received gestational insulin treatment. Three participants with type 2 DM withdrew, leaving a total of 49 PGDM pregnancies for statistical analyzes. Gestational age (GA) was determined using a vaginal probe (Vivid 7, GE Healthcare Vingmed Ultrasound, E8C, 8 MHz) at the first visit (around week 9), by measuring the crown rump length [24]. No fetal malformations were revealed by second-trimester routine scans in the study population. Information on first trimester maternal HbA_1C_, neonatal sex, birthweight, mode of delivery, Apgar score, cord-blood gases, and transfer to the neonatal ward was collected from clinical records. The results from the study group were compared with reference ranges established in the same research unit using identical methods, in a longitudinal study of 160 low-risk pregnancies [25, 26].

### Measurements

The ultrasound examinations were performed in each pregnancy at gestational weeks 24, 28, 32, and 36. All ultrasound measurements were performed by three observers (A.L., J.K. and C.E.) using an abdominal transducer (M4S, 2.0–4.3 MHz) ultrasound system (Vivid 7, GE Healthcare Vingmed Ultrasound, Horten, Norway). The sessions lasted maximum one hour and the thermal index was kept below 1.0.

The time-averaged maximum blood velocity (TAMXV) was measured in the umbilical vein, ductus venosus, left portal vein and portal vein (Fig 1). The angle of insonation was kept small, not exceeding 30° (median angle correction was 0, range 0–30°). At the same site perpendicular to the vessel wall, the inner vessel diameter (*D*) was measured at least three times in the umbilical vein, ductus venosus and portal vein. The mean *D* was used for the analyses (Fig 1). After identification of the vessel, the color Doppler was turned off and *D* was measured in magnified images. The techniques applied are described in detail elsewhere [25, 26].

Blood flow (*Q*, mL·min^–1^) was calculated by the formula *Q* = π ∙ (*D*/2)^2^ ∙ *h* ∙ TAMXV. The velocity profile parameter was *h* = 0.5 for the umbilical vein (UV) and the portal vein (PV) [26], *h* = 0.7 for the ductus venosus (DV) [27, 28]. Flow was normalized based on the estimated fetal weight (EFW) as *Q*/EFW (mL·min^–1^·kg^–1^) [29]. Umbilical venous liver flow (UV_liver_) was calculated as *Q*_UV liver_ = *Q*_UV_ − *Q*_DV_, total liver flow as *Q*_liver_ = (*Q*_UV_ − *Q*_DV_) + *Q*_PV_ and PV fraction (*F*_*PV*_) of the total venous supply to the liver was *F*_PV_ = 100% ∙ *Q*_PV_/*Q*_liver_.

### Statistics

The sample size was based on our previous studies in non-diabetic pregnancies, demonstrating significant associations between fetal growth patterns and variation in the venous liver circulation [7, 30]. We allowed for lower measurement success rates and possibly smaller effects in the PGDM group by increasing the number of participants from 30 to 50. It was not possible to perform a formal sample size calculation since there were no earlier reports on the effects of PGDM on fetal liver flow.

Multilevel regression analysis was used to model the mean and standard deviation values for the outcome variables according to gestational age. The absence of overlap of the 95% confidence intervals of the mean indicated a statistically significant difference between the PGDM group and the reference values [25, 26, 31]. In addition, *z*-scores for means of outcome variables in the study population were compared with the reference group using the independent-samples *t*-test, with a significance cutoff of *p*≤0.05. The populations were also stratified for gestational age (GA </≥ 30 weeks), and independent sample *t*-tests comparing mean *z*-scores were performed to test differences between PGDM and low-risk pregnancies before and after 30 weeks of gestation. The relations between maternal first-trimester HbA_1c_ and left portal vein flow velocity, portal venous flow, and portal venous shunt fraction *z*-scores after 30 gestational weeks were assessed using multilevel regression analysis. The statistical analyses were performed with the Statistical Package for the Social Sciences (version 24, SPSS, Chicago, IL) and the MLWin program (version 2.35, Centre of Multilevel Modeling, University of Bristol, UK).

## Results

The characteristics of the study population are described in Tables 1 and 2. The median gestational age at birth was lower and birthweights were higher in the study group than in the reference population [31]. In the study group, 19 (39%) of the neonates were macrosomic (birthweight >90^th^ percentile) and 3 (6%) were small for gestational age (<10^th^ percentile) [29] (Tables 1 and 2).

**Table 1:** Maternal characteristics and outcomes in 49 pregnancies with pregestational diabetes mellitus.

	**Number**	**Percent**
Type 1 DM	44	89.8
Type 2 DM	5	10.2
Maternal diabetic complications or condition		
- Retinopathy	9	18.4
- Nephropathy	1	2.0
- Hypothyroidism	9	18.4
- Chronic hypertension	7	14.3
Preeclampsia	3	6.1
Preterm birth	15	30.6
Induction of labor	30	61.2
Normal delivery	20	40.8
Operative vaginal delivery	7	14.3
Cesarean section	22	44.9
- Elective	9	18.4
- Acute	13	26.5
	**Median**	**Range**
Maternal age (years)	31	23 to 42
Pre-pregnancy weight (kg)	70	57 to 113
Maternal weight gain	15.8	-5.0 to 33.1
Pre-pregnancy BMI	24.9	19.8 to 44.1
HbA_1c_ at inclusion (%)	6.7	4.9 to 12.0
Individual mean HbA_1c_† (%)	6.12	4.9 to 8.2

DM, diabetes mellitus; Preterm birth, gestational age <37 weeks

*acute cesarean section during labor

†mean of all HbA_1c_ measurements throughout each pregnancy

**Table 2:** Neonatal characteristics and outcomes in pregnancies with pregestational diabetes mellitus.

	**Median**	**Range**
Gestational age at delivery (weeks+days)	38+4	27+6 to 40+5
Birthweight (g)	3695	990 to 5990
Birthweight *z*-score	0.93	–2.15 to 5.82
Umbilical artery		
- pH	7.24	6.92 to 7.34
- pCO_2_ (kPa)	7.88	5.80 to 12.40
- pO_2_ (kPa)	2.19	1.16 to 3.47
- Base deficit (mmol·L^–1^)	–2.11	–13.36 to 1.00
- Lactate (mmol·L^–1^)	4.70	2.00 to 14.40
Umbilical vein		
- pH	7.30	6.89 to 7.44
- pCO_2_ (kPa)	6.10	4.20 to 15.30
- pO_2_ (kPa)	3.29	0.25 to 5.68
- Base deficit (mmol·L^–1^)	–2.36	–10.98 to –0.15
- Lactate (mmol·L^–1^)	3.50	1.80 to 12.80
Erythrocyte volume fraction	0.63	0.52 to 0.76
	**Number**	**Percent**
Male sex	25	51%
Operative delivery for intrapartum fetal distress	13	26.5%
Metabolic acidosis at birth*	1	2%
5-min Apgar score <7	1	2%
Neonatal intensive care	20	40.8%
Perinatal death†	1	2%
Malformation‡	2	4%

* Metabolic acidosis defined as an umbilical arterial pH of <7.0 and a base deficit of >12.

† Intrauterine fetal death at gestational week 36. Autopsy showed UV thrombosis and signs of acute asphyxia.

‡ One neonate with sagittal craniosynostosis and one with congenital heart defect (anomalous left coronary artery from the pulmonary artery)

The left portal vein and portal vein blood velocity, and the portal vein diameters, were successfully measured in 94.4%, 70.9% and 55.9% of 179 examination sessions, respectively. Further, portal venous flow was calculated in 52.5%, total venous liver flow (Q_liver_) in 42.5%, and the portal venous fraction of the total venous liver flow in 42.5% of the sessions. The success rate for the umbilical vein and ductus venosus measurements have been published earlier [14].

**Fig 2:**
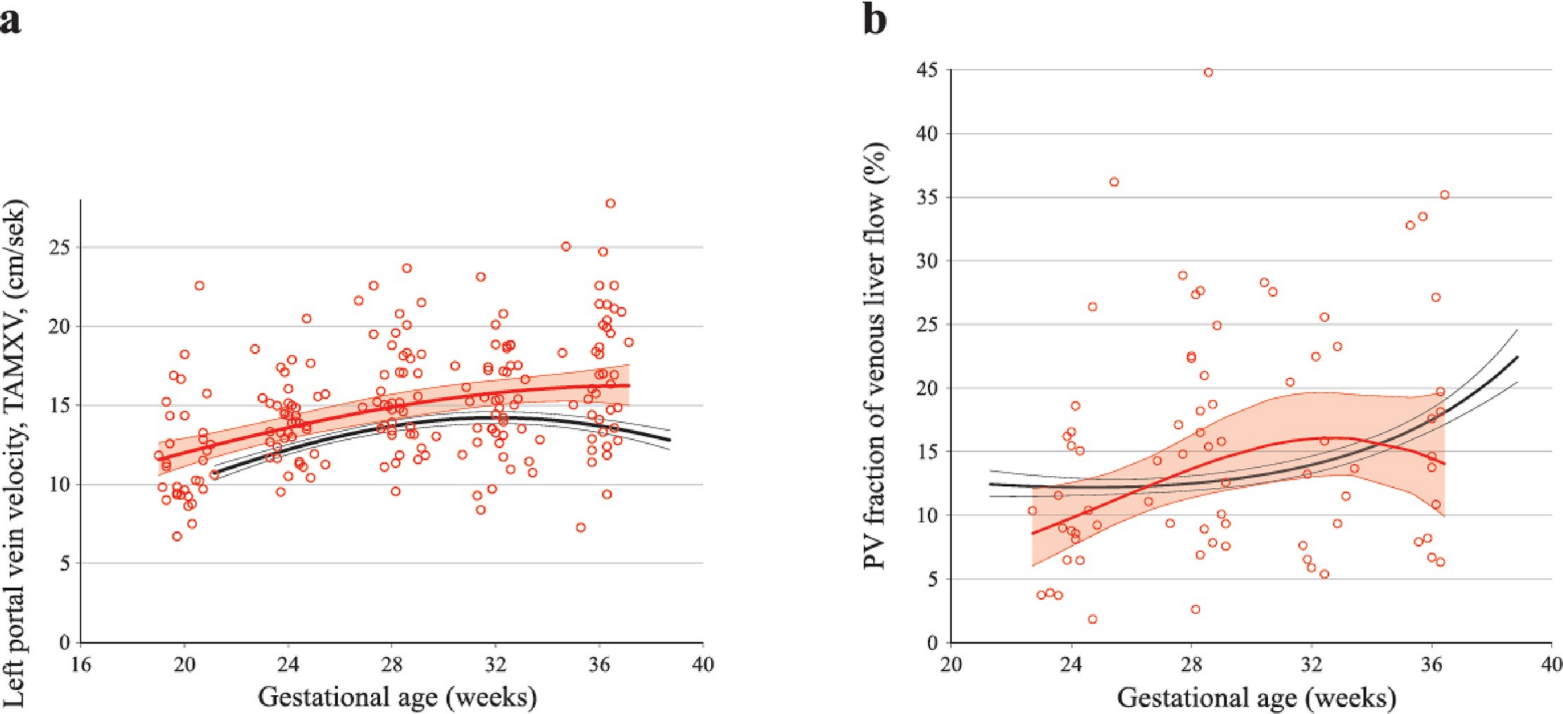
Longitudinal observations of left portal vein blood velocity and portal venous fraction in PGDM and low-risk pregnancies. Left portal vein blood velocity (TAMXV) as marker of the watershed between portal and umbilical contribution to fetal venous liver flow **(a),** and the portal fraction (%) of total venous volume **(b)** in 49 pregnancies with pregestational diabetes (PGDM; red circles and lines) compared with reference values from a low-risk population (black lines) presented with mean (thick lines) and 95% confidence interval (thin lines).

The mean left portal vein flow velocity in the PGDM group was significantly higher than the reference values, both before and after 30 weeks (Table 3, Fig 2A).

**Table 3:** Fetal venous liver blood flow in pregnancies complicated by pregestational diabetes mellitus compared with reference values from a low risk population.

Parameter	Popu-lation	n	Mean *z*-score	*p*	*GA <30 Mean z-score*	*GA<30p*	*GA≥30 Mean z-score*	*GA≥30p*
			(95% CI)		(95% CI)		(95% CI)	
LPV velocity (cm/s)	Ref.	537	0.003	<0.001	0.011	<0.001	-0.005	<0.001
			(-0.09–0.09)		(-0.11–0.13)		(-0.12–0.12)	
	PGDM	201	0.639		0.675		0.575	
			(0.49–0.79)		(0.46–0.89)		(0.29–0.86)	
PV flow (mL·min^–1^)	Ref.	547	0.011	0.052	0.016	0.005	0.006	0.131
			(-0.09–0.11)		(-0.10–0.13)		(-0.12–0.13)	
	PGDM	93	0.272		0.796		-0.466	
			(0.03–0.52)		(0.26–1.32)		(-1.07–0.14)	
Normalized PV flow (mL·min^-1^·kg^-1^)	Ref.	547	0.007	0.002	0.022)	0.821	0.009	0.002
			(-0.10–0.11)		(-0.09–0.13		(-0.14–0.12)	
	PGDM	93	-0.418		0.089		-1.132	
			(-0.67 - -0.17)		(-0.49–0.67)		(-1.80 - -0.46)	
Total venous liver flow, Q_liver_ (mL·min^–1^)	Ref.	514	-0.005	<0.001	-0.008)	0.001	-0.001	0.881
			(-0.10–0.09)		(-0.13–0.11		(-0.13–0.13)	
	PGDM	75	0.507		0.847		-0.045	
			(0.26–0.75)		(0.39–1.30)		(-0.63–0.54)	
Normalizedvenous liver flow (mL·min^–1^·kg^–1^)	Ref.	473	0.010	0.479	0.007	0.342	0.033	0.047
			(-0.09–0.11)		(-0.13–0.11)		(-0.11–0.17)	
	PGDM	75	-0.085		0.195		-0.538	
			(-0.33–0.16)		(-0.21–0.60)		(-1.08–0.01)	
PV fraction of total venous liver flow (%)	Ref.	511	0.004	0.645	-0.002	0.909	0.012	0.550
			(-0.09–0.10)		(-0.12–0.11)		(-0.12–0.14)	
	PGDM	75	-0.098		0.028		0.217	
			(-0.35–0.16)		(-0.49–0.54)		(-0.46–0.89)	
UV liver flow, Q_UV liver_ (mL·min^–1^)	Ref.	558	0.00	0.002	-0.02	<0.001	0.01)	0.952
			(-0.09–0.10)		(-0.13–0.09)		(-0.11–0.14	
	PGDM	122	0.364		0.65		0.00	
			(0.16–0.57)		(0.23–1.06)		(-0.37–0.38)	
Normalized UV liver flow (mL·min^–1^·kg^–1^)	Ref.	558	0.004	0.229	-0.05	0.630	0.03)	0.049
			(-0.09 –-0.10)		(-0.17–0.07)		(-0.09–0.15	
	PGDM	122	-0.131		0.03		-0.33	
			(-0.33–0.06)		(-0.35–0.41)		(-0.67–0.00)	

PGDM, pregestational diabetes mellitus; Ref., low-risk reference group [25, 26]; n, number of observations; CI, confidence interval for the mean *z*-score; *p*, probability value; GA, gestational age (weeks)—before and after 30 weeks; LPV, Left portal vein; PV, portal vein; Q_liver_, total venous liver flow; PV fraction (%) = (PV flow/Total liver flow)·100; Q _UV liver_, umbilical venous flow to the liver

**Fig 3:**
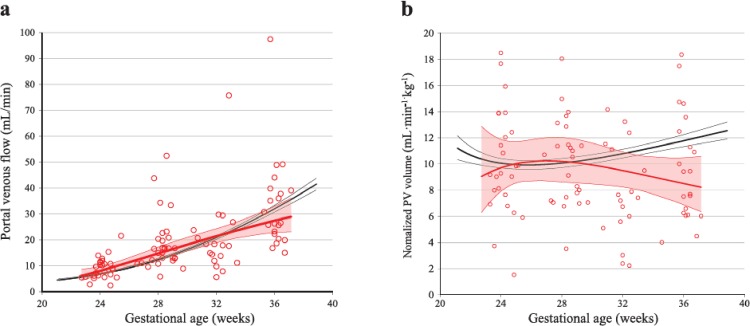
Longitudinal observations of portal venous flow in PGDM and low-risk pregnancies. Portal venous flow **(a)** and normalized portal venous flow **(b)** in 49 pregnancies with pregestational diabetes (PGDM; red circles and lines) compared with reference values from a low-risk population (black lines), with mean (thick lines) and 95% confidence-interval (thin lines).

The mean portal venous flow in the PGDM pregnancies was not significantly different from the reference values over the study period as a whole, but was significantly higher for the period before 30 weeks of gestation, and the development after 30 weeks was blunted compared with the reference values (Fig 3A). When normalized for EFW, the overall mean portal venous flow was significantly smaller in PGDM, mainly due to reduced flow after 30 weeks of gestation (Table 3 and Fig 3B).

**Fig 4:**
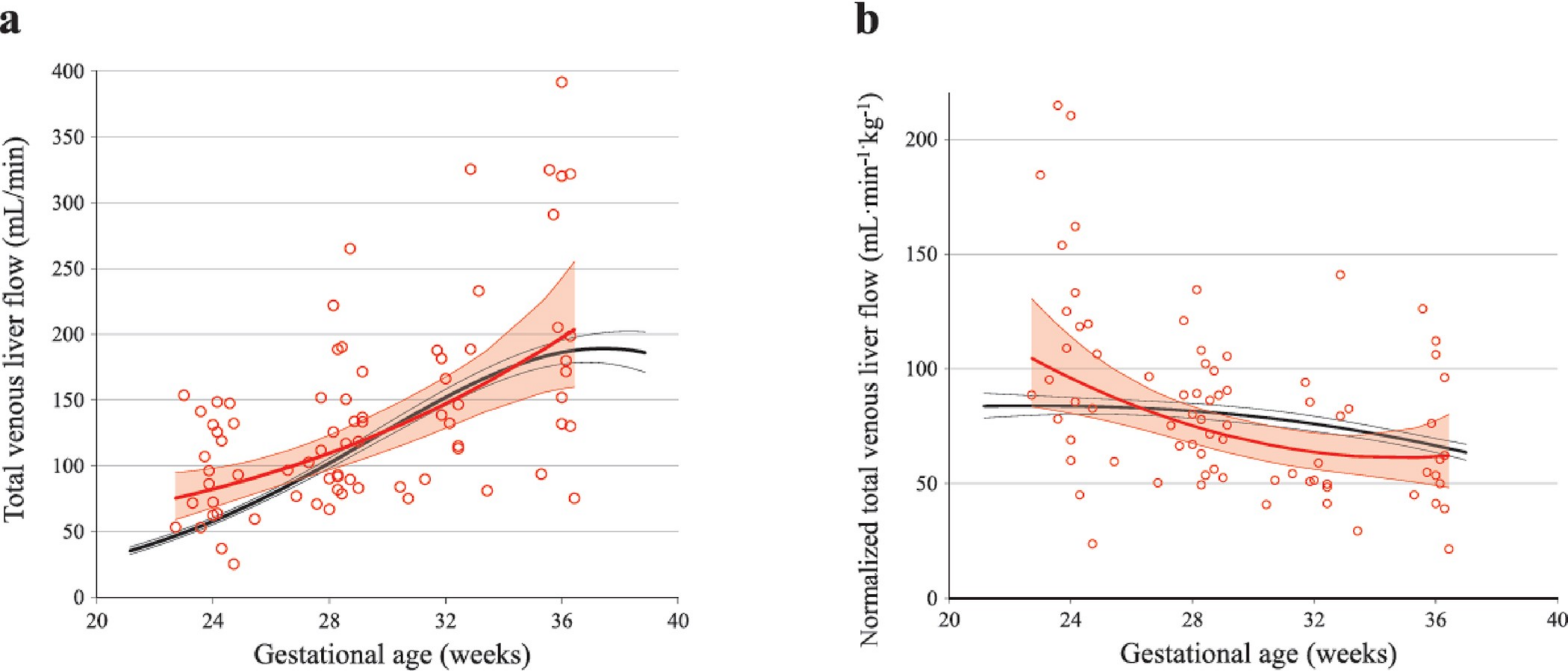
Longitudinal observations of total venous supply to the fetal liver in PGDM and low-risk pregnancies. Total venous liver flow **(a)** and the correspondingly normalized flow values **(b)** in 49 pregnancies with pregestational diabetes mellitus (PGDM; red circles and lines) compared with reference values from a low-risk population (black lines) presented with mean (thick lines) and 95% confidence interval (thin lines).

The total venous supply to the fetal liver (Q_liver_) was larger in PGDM pregnancies (Table 3), mainly due to high volumes in the second trimester (Fig 4A). When normalized for EFW, the overall mean total venous liver flow in the PGDM group did not differ from that of the reference values but was significantly smaller after 30 weeks and with a different trajectory of the mean curve (Table 3 and Fig 4B).

In the study group, the mean portal venous fraction for all observations through pregnancy did not differ from the low-risk group (Table 3). However, the curve describing mean portal venous fraction had an inverted U-shape in PGDM fetuses, the opposite of that in the reference group, where the portal venous fraction increased after week 33 (Fig 2B).

**Fig 5:**
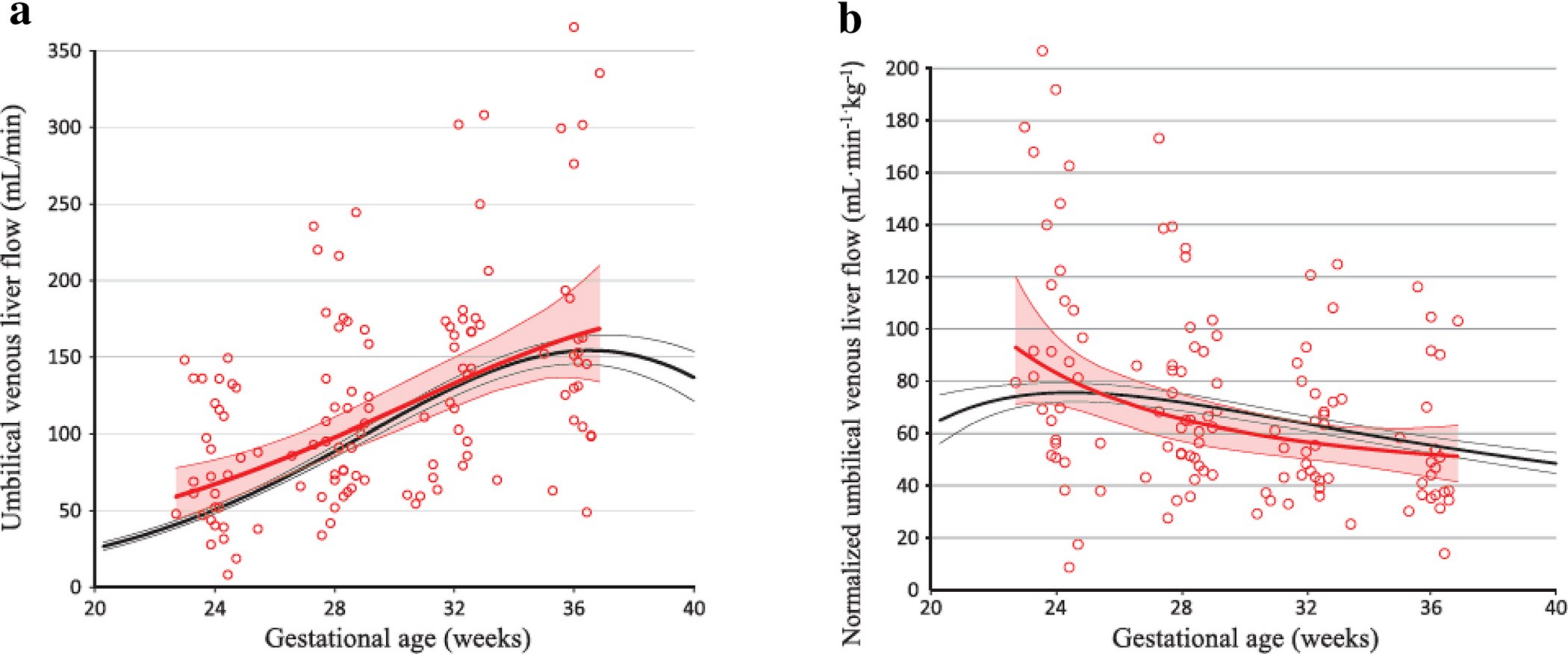
Longitudinal observations of the umbilical venous supply to the fetal liver in PGDM and low-risk pregnancies. Umbilical venous liver flow **(a)** and the correspondingly normalized flow values **(b)** in 49 pregnancies with pregestational diabetes mellitus (PGDM; red circles and lines) compared with reference values from a low-risk population (black lines) presented with mean (thick lines) and 95% confidence interval (thin lines).

The overall mean umbilical venous liver flow (Q _UV liver_) was higher in PGDM pregnancies compared with the reference, mainly due to the high flows before 30 weeks of gestation (Fig 5A). However, when normalized for EFW, the overall mean umbilical venous liver flow was not different from low-risk pregnancies (Table 3), but the trajectory of the flow development tended to be different in PGDM pregnancies with borderline significantly lower flow after 30 weeks (Table 3, Fig 5B).

**Fig 6:**
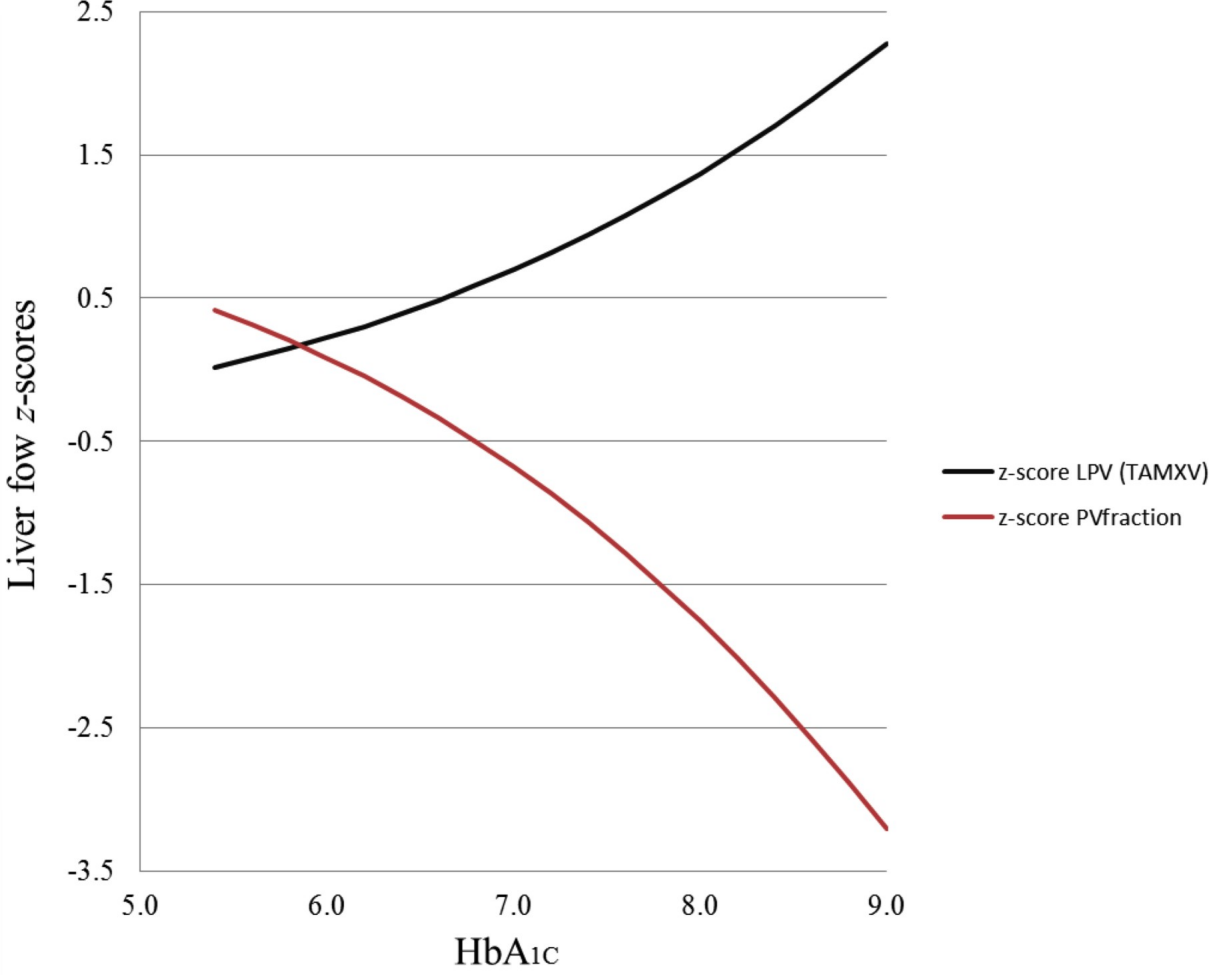
Fetal liver blood flow and relation to HbA_1C_. Relations between z-scores of the time-averaged maximum left portal vein (LPV) flow velocity (TAMXV) and portal vein (PV) fraction, and first-trimester HbA_1c_.

The *z*-scores for left portal vein blood velocity were positively related to first trimester HbA_1C_ and correspondingly, the *z*-scores for portal venous fraction were negatively related to first trimester HbA_1C_ (Fig 6). There was no relation between HbA_1C_ and portal venous or total venous liver flow.

**Fig 7:**
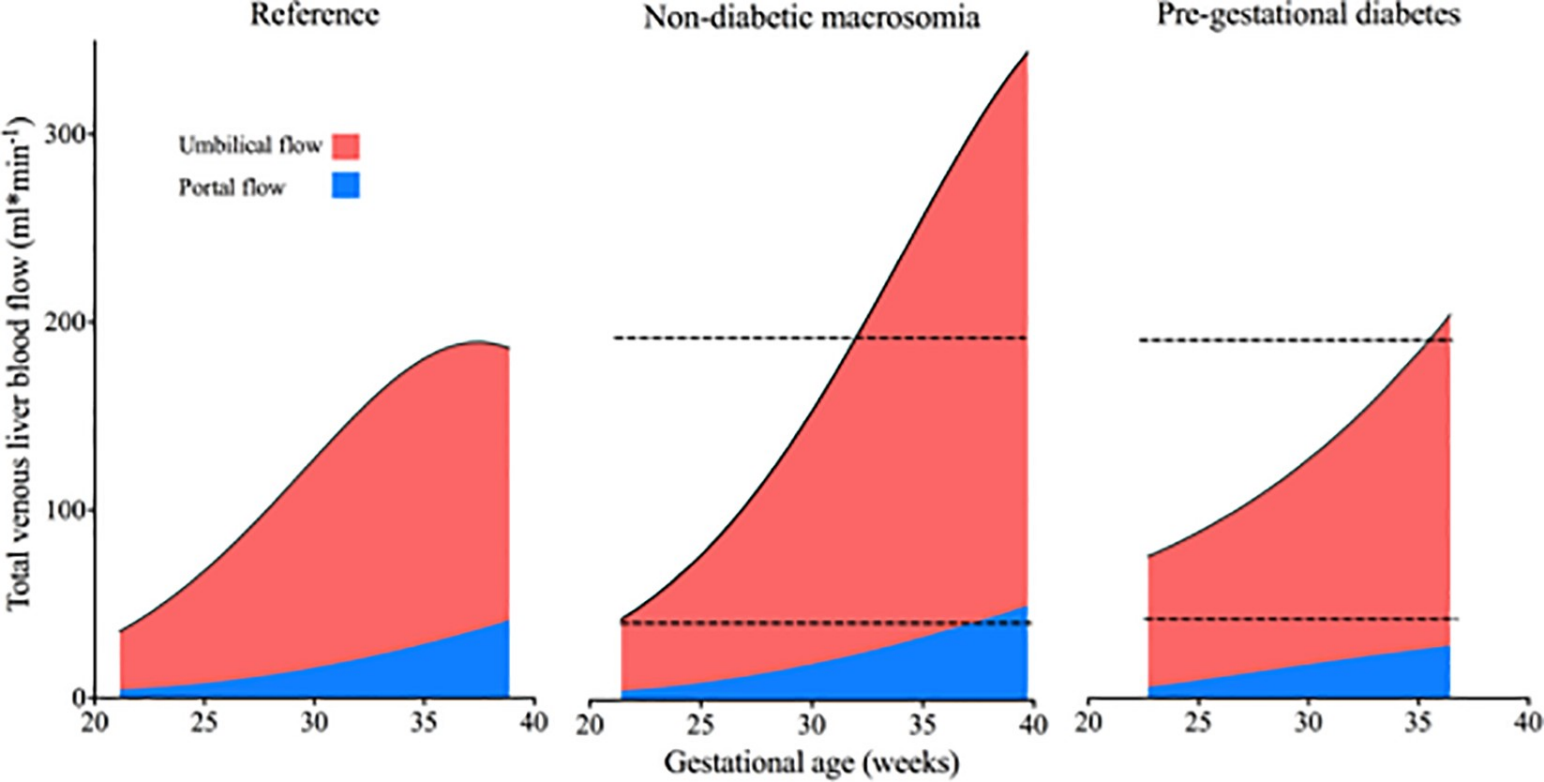
Fetal venous liver flow development in pregnancies with low risk, macrosomia and PGDM. The fetal venous liver flow in three different populations: a low-risk population (physiological venous liver flow during the last weeks of pregnancy; dotted lines), fetal macrosomic growth *without* maternal diabetes, and pregnancies with pregestational diabetes mellitus (PGDM, the present study population).

Since 39% of the neonates in our PGDM group were macrosomic, we compared the development of the total venous liver, umbilical and portal flows in low-risk, non-diabetic macrosomic and PGDM pregnancies, to illustrate the different flow patterns (Fig 7).

We compared the mean *z*-scores in the T1DM group with the reference values for LPV velocity, PV flow, normalized PV flow, total venous liver flow, normalized venous liver flow and UV liver flow (S1 Table). Excluding T2DM participants from the PGDM population did not significantly change the results, except for PV flow which then became borderline significantly higher compared with the reference values (*p* = 0.046).

## Discussion

In pregnancies complicated with PGDM, the fetal liver perfusion with nutritious umbilical blood from the placenta was prioritized (Table 3, Figs 1 and 2A). This effect was graded according to the maternal HbA1c level (Fig 6) and was associated with correspondingly accelerated fetal growth during the 2^nd^ trimester. However, the blunted umbilical flow development during the 3^rd^ trimester seemed to cause an increasing mismatch between growth and blood supply (Table 3, Figs 2–5).

The venous supply to the liver is radically different in fetal and postnatal life, with contributions from both the umbilical (≥80%) and the portal vein (≤20%) (Figs 1 and 7) [26]. Thus, the umbilical vein is the principal source of fetal liver blood supply [31], and this umbilical venous flow to the liver is augmented in diabetic pregnancies (Table 3, Fig 5A). Such increased delivery of oxygen and nutrient rich umbilical venous blood to the liver, is thought to be instrumental in the development of macrosomia [8].

The left portal vein connects the umbilical vein with the portal circulation and directs umbilical venous blood to the right lobe of the liver (Fig 1). Blood flow in the left portal vein is regulated by catecholamines [32] and maternal glucose levels [10]. Measurement of the left portal vein velocity alone provides a simple method for gauging the umbilical/portal watershed and for assessment of intrahepatic venous redistribution in compromised fetuses [25]. In the present study, the mean left portal vein velocity was higher in PGDM pregnancies than the reference values, throughout the second half of pregnancy (Fig 2A). This signifies increased prioritization of umbilical blood flow to the right liver lobe and is known to induce liver growth, increased production of IGF-1 and -2 and in turn, differential organ growth [8, 9].

The portal contribution to the venous liver perfusion was higher in PGDM than the reference group before 30 weeks (Table 3), but the portal venous flow did not keep up with fetal growth later in pregnancy (Fig 3B). This corroborates a study of Olofsson *et al*., showing that blood flow to the lower extremities was prioritized at the expense of visceral blood flow during the third trimester, in pregnancies with type 1 DM [33]. In addition, lower portal venous return could be a result of reduced fetal swallowing or intestinal activity in diabetic pregnancies [34]. We hypothesize that the increased umbilical venous flow to the right liver lobe observed in our study, may induce accelerated fetal growth that is less supported by umbilical venous supply at the end of pregnancy, and without any increase of portal blood flow to the liver. Higher umbilical venous flow to the right liver lobe, as found in our study, could also influence liver gene expression [15], fetal body composition [11] and possibly later health [20].

In the present study, 39% of the newborns had a birthweight > 90^th^ percentile for gestational age (Table 2). Fetal macrosomia in PGDM is different from that in non-diabetic pregnancies, with disproportionate fetal growth expressed as a higher ponderal index [35]. In a study of macrosomic fetuses *without* maternal diabetes, the umbilical venous perfusion [12], left portal venous flow, portal and total venous liver flow, were all increased during the second half of pregnancy [7], even when corrected for fetal weight. Similarly, in the present study, high umbilical venous flow [14], correspondingly low placental impedance [36] and increased portal blood flow permit an up-regulation of liver flow before 30 gestational weeks (Table 3, Figs 2–5). In contrast, after 30 weeks gestation, fetuses of diabetic mothers had reduced portal and total venous liver flow when normalized for fetal weight, while in non-diabetic macrosomic fetuses no restriction in venous blood flow to the liver was observed (Table 3, Fig 7).

It is known that during placental compromise associated with fetal growth restriction, shunting through the ductus venosus is prioritized at the expense of the umbilical venous liver flow [37, 38]. This leads to reduced liver size that increasingly depends on the low-oxygenated portal flow. In PGDM pregnancies however, the increased risk of chronic hypoxemia, acidosis, and perinatal death in the last weeks of gestation [39–41] follows relatively greater umbilical supply during the 2^nd^ trimester (Table 3). The liver received umbilical blood at the expense of flow through the ductus venosus [14]. Towards the end of pregnancy, PGDM fetuses outgrew their supply of umbilical venous blood and did not maintain portal flow corresponding to their weight (Figs 2 and 3). Although being at risk of relative hypoxia, the re-distribution mechanisms well-known in fetal growth restriction did not seem to operate.

The strengths of this study are its prospective longitudinal design, involving an unselected group of PGDM pregnancies, and identical and validated ultrasound and Doppler methods applied to the reference population [42]. Low intra- and inter-observer variation has been demonstrated for measurements of ductus venosus flow velocities [43], and almost identical results for umbilical venous flow were achieved by different investigators using the same technique for ultrasound measurement and blood flow calculation [26, 44, 45]. The success rate for measurements varied from 93.4% for left portal vein blood velocity to 52.5% for portal venous flow, the latter being lower than in the reference population [26]. This was mainly due to difficult examination conditions caused by high BMI in our study group. Although there was no inter-group difference in liver flow between PGDM participants with BMI< or ≥30, a selection bias cannot entirely be ruled out. Because the BMI was borderline significantly higher in the missing compared with the complete data group (tested by independent sample *t*-test, mean BMI *z*-score in the Q_UV liver_ missing data vs. non-missing data groups were 1.47 and 1.13 respectively, *p* = 0.07) this could introduce selection of a leaner PGDM population for the estimation of umbilical venous liver flow. However, such a selection is expected to reduce rather than augment the differences between the study- and the reference populations. Also, wider confidence intervals in the study group compared with the reference group warrant a cautious interpretation of the findings.

Including women with type 1 and type 2 DM in one study group may represent a limitation, since these conditions differ in many respects. Our goal was however, to study fetal flow and growth in pregnancies with PGDM. Our population was not large enough to answer the question of whether fetal venous liver circulation is different in pregnancies with type 1 or type 2 DM. Nevertheless, when women with type 2 DM were excluded the findings remained significant in the type 1 DM group (S1 Table).

## Conclusion

Maternal diabetes is associated with adverse consequences in the offspring [46], including macrosomia and metabolic syndrome [47], but the underlying mechanisms are not established. Fetal liver blood flow is linked to fetal growth, and we showed that flow is related to maternal blood glucose in the first trimester in PGDM pregnancies. However, the relatively greater liver perfusion in PGDM pregnancies before 30 weeks was not maintained in late gestation, possibly leading to mismatch between fetal growth and nutrient supply, and later effects on health.

## Supporting information

S1 Table Fetal venous liver blood flow in pregnancies complicated by type 1 diabetes mellitus compared with a low risk reference population. Ref., low-risk reference group; n, number of observations; CI, confidence interval for the mean *z*-score; *p*, probability value; LPV, Left portal vein; PV, portal vein; Q_liver_, total venous liver flow; UV liver flow, umbilical venous flow to the liver.
(DOCX)
