# Supplementary material for: Altered development of fetal liver perfusion in pregnancies with pregestational diabetes
Source: PLoS One. 2019 Mar 13;14(3):e0211788. doi: 10.1371/journal.pone.0211788 (PMC6415794; doi:10.1371/journal.pone.0211788)
Supplement: S1 Table — Ref., low-risk reference group; n, number of observations; CI, confidence interval for the mean z-score; p, probability value; LPV, Left portal vein; PV, portal vein; Qliver, total venous liver flow; UV liver flow, umbilical venous flow to the liver. (DOCX) [file pone.0211788.s001.docx]

**Supporting information**

**S1 Table**

**Fetal venous liver blood flow in pregnancies complicated by type 1 diabetes mellitus compared with a low risk reference population**

| **Parameter** | **Population** | **n** | **Mean**  ***z*-score**  **(CI of mean)** | ***p*** |
| --- | --- | --- | --- | --- |
| LPV velocity  (cm/s) | Ref. | 553 | 0.004  (-0.86 – 0.09) | *<0.001* |
|  | Type 1DM | 179 | 0.691  (0.29 – 0.87) |  |
| PV flow  (mL·min^–1^) | Ref. | 558 | 0.017  (-0.87 – 0.09) | 0.046 |
|  | Type 1DM | 86 | 0.297  (-0.05 – 0.99) |  |
| Normalized  PV flow  (mL⋅min^-1^·kg^-1^) | Ref. | 558 | 0.011  (-0.09 – 0.08) | *0.002* |
|  | Type 1DM | 86 | -0.450  (-0.88 – 0.26) |  |
| Total venous  liver flow,  Q_liver_ (mL·min^–1^) | Ref. | 525 | -0.002  (-0.10 – 0.08) | *<0.001* |
|  | Type 1DM | 69 | 0.570  (0.14 – 0.92) |  |
| Normalized  venous  liver flow  (mL·min^–1^·kg^–1^) | Ref. | 528 | -0.012  (-0.09 – 0.09) | 0.598 |
|  | Type 1DM | 69 | -0.087  (-0.48 – 0.21) |  |
| UV liver flow,  Q _UV liver_  (mL·min^–1^) | Ref. | 555 | -0.006  (-0.12 – 0.06) | *<0.001* |
|  | Type 1DM | 111 | 0.435  (-0.09 – 0.76) |  |

Ref., low-risk reference group; n, number of observations; CI, confidence interval for the mean *z*-score; *p*, probability value; LPV, Left portal vein; PV, portal vein; Q_liver_, total venous liver flow; UV liver flow, umbilical venous flow to the liver; Type 1 DM, type 1 diabetes mellitus
